# Pharmacokinetic and pharmacodynamic studies of nicotine in rat brain: a simultaneous investigation of nicotine metabolites and the release of neurotransmitters *in vivo*

**DOI:** 10.3389/fchem.2023.1275478

**Published:** 2023-10-23

**Authors:** Lulu Guo, Jian Mao, Qidong Zhang, Wu Fan, Dingzhong Wang, Zhonghao Li, Jiaqiang Huang, Jianping Xie

**Affiliations:** ^1^ Department of Nutrition and Health, Beijing Advanced Innovation Center for Food Nutrition and Human Health, China Agricultural University, Beijing, China; ^2^ Beijing Life Science Academy, Beijing, China; ^3^ Food Laboratory of Zhongyuan, Zhengzhou University, Zhengzhou, China

**Keywords:** nicotine, neurotransmitters, brain metabolism, online-microdialysis, UHPLC-HRMS/MS

## Abstract

**Introduction:** The body’s ability to metabolize nicotine and the disposition of nicotine in the brain are important determinants of its exposure. Limited knowledge about the near real-time changes of neurochemicals during the brain nicotine metabolic process hinders the recognition of its multiple neuropharmacological effects.

**Methods:** An online microdialysis coupled with UHPLC-HRMS/MS method for the *in vivo* multi-analysis of nicotine metabolites and several neurotransmitters in rat brain was developed. Whether the systemic modulation of metabolic enzyme CYP2B would modulate nicotine pharmacokinetics and local neurochemical effects was further investigated.

**Results:** The dynamic profiles of over 10 nicotine metabolites and neurotransmitters were simultaneously obtained after a single injection of nicotine (2 mg·kg^−1^, i.p.) using the new method. Proadifen pretreatment (50 mg·kg^−1^·d^−1^, i.p., 4 days) caused significant inhibition of brain CYP2B1 activity. When exposed to nicotine, the brain *C*_max_ of nicotine was 1.26 times higher and the levels of nicotine metabolites, nornicotine, and nicotine-*N*-oxide, were decreased by 85.3% and 34.4% in proadifen-pretreated rats. The higher level of brain nicotine induced a greater release of dopamine, serotonin, glutamate, and γ-amino-butyric acid in the nucleus accumbens. The concentrations of nicotine and dopamine were positively correlated, and the average levels of γ-amino-butyric acid and serotonin were 2.7 and 1.2 times higher, respectively, under the inhibition of nicotine metabolism.

**Discussion:** These results demonstrated that inhibiting nicotine metabolism in rats can enhance the residence of brain nicotine and its local neurotransmitter effects. The metabolic activity of nicotine under different physiological conditions could regulate nicotine’s bioavailability and its resulting pharmacology.

## 1 Introduction

Nicotine (Nic) is a principal bioactive ingredient in cigarettes and the component most associated with tobacco dependence (Tanner et al., 2015; Xiao et al., 2020). The development and persistence of tobacco dependence are due to the reward effect produced by activating nicotine acetylcholine receptors (nAChRs) in the mesolimbic reward circuitry (Tapper et al., 2004; Changeux, 2010; Henderson et al., 2017; Chen et al., 2022). In addition to its strong addictive potential, extensive studies showed that Nic is a potential drug for the amelioration of ulcerative colitis, Alzheimer’s disease, and Parkinson’s disease (Quik et al., 2012; Quik et al., 2012). A rapid elevation of Nic in the brain may be the first choice for the treatment of Nic dependence; however, for the amelioration of Alzheimer’s disease and Parkinson’s disease, maintaining an effective Nic level in the brain for a long time may be a better approach (Alsharari et al., 2014). The bioavailability and pharmacokinetics of Nic were found to be affected by its molecular form and metabolic enzyme activity (Hwa Jung et al., 2001). The multiple physiological effects of Nic are mainly because Nic can potently regulates the release of neurochemicals and even the metabolism of neurotransmitters, including dopamine (DA), γ-amino-butyric acid (GABA), glutamate (Glu), serotonin (5-HT), and acetylcholine (Ach), within various mesocorticolimbic structures, such as the ventral tegmental area (VTA), nucleus accumbens (NAc), and prefrontal cortex (Grieder et al., 2019; Vivekanandarajah et al., 2019; Jadzic et al., 2021; Nguyen et al., 2021; Pietilä et al., 1996). Therefore, investigating the release information of neurotransmitters in the brain during Nic exposure is an effective approach to studying the multiple effects of Nic.

The local biodegradation and metabolic process of Nic is an important determinant of its exposure in the brain. Even though peripheral analysis of Nic and its metabolites by the more sensitive method HPLC-MS have been studied extensively in biological fluids, the metabolism of Nic in the central nervous system (CNS) has been given little attention (Rangiah et al., 2011; Piller et al., 2014; Mao et al., 2015). Similar to peripheral system in the liver and blood, Nic has been confirmed to be oxidized, *N*-demethylated, and hydroxylated in the CNS (Xu et al., 2019), which generates cotinine (Cot), nicotine-*N*-oxide (NNO), nornicotine (NNic), norcotinine (NCot), trans-3′-hydroxy-cotinine (OH-Cot), 4-oxo-4-(3-pyridyl)-butanoic acid (OxPyBut), and 4-hydroxy-4-(3-pyridyl)-butanoic acid (HyPyBut). Several studies showed that some Nic metabolites also have pharmacological activity and may contribute to the multiple effects of Nic exposure. For example, the main metabolite Cot, which acts as a weak agonist of nAChRs, was considered to actively regulate the cholinergic, DAergic, and 5-HTergic systems, and promote synaptic plasticity and stress resilience (Mendoza et al., 2018; Oliveros-Matus et al., 2020). Other studies have shown that regulation of the Nic metabolism changed its bioavailability and pharmacology. Inhibition of mouse CYP2A5 increased the bioavailability of Nic and prolonged the duration of analgesia and hypothermia induced by Nic (Alsharari et al., 2014). Furthermore, in rats and monkeys, continuous treatment with Nic increased the levels of the Nic metabolizing enzyme CYP2B in the cerebrum, which decreased brain Nic levels, altered the reinforcing effects, and thereby increased withdrawal symptoms (Garcia et al., 2015). However, it is unknown how the changes of physiological effects caused by different brain Nic levels were clearly related to the release of neurotransmitters. Therefore, it is necessary to synchronously investigate the brain distribution, metabolic characteristics, and the related neurotransmitter effects resulting from Nic exposure in the CNS.

Synchronous investigation of brain Nic pharmacokinetics and the related local changes of neurotransmitters have been given little attention due to the difficulty of obtaining the brain samples *in vivo* and a lack of effective detection methods. The greatest obstacle is that the different polarity, unstable chemical properties, and low concentration in biological matrices hinders the quantitative determination of most neurotransmitters and some Nic metabolites (Jha et al., 2018; Olesti et al., 2019; El-Sherbeni et al., 2020; Zhou et al., 2020). Microdialysis coupled with analytical techniques such as HPLC-MS/MS has become a workhorse and a state-of-the-art technique to assess the *in vivo* changes of extracellular compounds, and has been applied extensively in neuroscience, pharmacokinetics, and pharmacodynamics (Nandi and Lunte, 2009; Zestos et al., 2019; Shi et al., 2014). However, the off-line microdialysis based analytical systems still suffer from some drawbacks, such as unexpected contamination, evaporation, or degradation of the analytes during sample storage and transfer. Online analysis is a better alternative that largely circumvents these problems (Wang et al., 2015). The direct coupling of microdialysis with HPLC-MS/MS enables the integration of *in vivo* minimally invasive sampling with high temporal resolution analysis for continuous monitoring of drug pharmacological effects. Meanwhile, derivatization regents such as benzoyl chloride, N-Boc-l-tryptophan hydroxysuccinimide ester, and (5-N-succinimidoxy-5-oxopentyl) triphenylphosphonium bromide have been employed to improve the separations and increase the detection sensitivity of neurotransmitter by LC-MS (Song et al., 2012; Greco et al., 2013; Zhang et al., 2014). Here, a precolumn “one-step” derivatization-based online microdialysis coupled with ultra-high performance liquid chromatography-high resolution tandem mass spectrometry (UHPLC-HRMS/MS) was developed, which integrated the *in vivo* sampling with online injection for the simultaneous detection of Nic metabolites and several neurochemicals. Relevant information on brain Nic pharmacokinetics and neurotransmitter release was effectively obtained in different rat models (i.e., normal group and enzyme inhibition group), which provided an in-depth understanding of Nic neuropharmacology under different physiological conditions.

## 2 Materials and methods

### 2.1 Chemicals and materials

Nicotine and its metabolites standards were purchased from Toronto Research Chemicals (North York, Canada) (−) nicotine (Nic), (−) cotinine (Cot), (*R*, *S*)- nornicotine (NNic), (*R*, *S*)-norcotinine (NCot), *trans*-3′-hydroxycotinine (OH-Cot), (1′*S*, 2′*S*)-nicotine-1′-oxide (NNO), (*S*)-cotinine-*N*-oxide (CNO), 4-oxo-4-(3-pyridyl)-butanoic acid (OxPyBut), 4-hydroxy-4-(3-pyridyl)-butanoic acid (HyPyBut), (1′*S*, 2′*S*)-nicotine-1′-oxide-*d*_3_ (NNO-*d*_3_), and (*R*, *S*)- nornicotine-*d*_4_ (NNic-*d*_4_). Dopamine hydrochloride (DA), 3,4-dihydrophenylacetic acid (DOPAC), homovanillic acid (HVA), serotonin hydrogenoxalate (5-HT), 5-hydroxyindole-3-acetic acid (5-HIAA), γ-amino-butyric acid (GABA), glutamate (Glu), and Acetylcholine chloride (Ach) were purchased from Sigma-Aldrich (St Louis, MO, USA) unless otherwise noted. Distilled deionized water (>18.2 MΩ cm^-1^ at 25 °C) was obtained from Thermo Scientific GenPure (Thermo-Fisher Scientific, Stockland, Niederelbert, Germany). Isoflurane was purchased from RWD Life Science Co., Ltd. (Shenzhen, China).

The stock solution of 10 g L^-1^ Nic and its metabolites were prepared by HPLC grade acetonitrile. 5 g L^-1^ DA, DOPAC, HVA, 5-HT, 5-HIAA, GABA, Glu, and Ach were prepared with acetonitrile-water 1:1 (*v/v*) with 1% hydrochloric acid to preserve the stability of neurotransmitters (Saracino et al., 2015; Tufi et al., 2015). The intermediate solution of Nic and its metabolites and neurotransmitters (1 g L^-1^) were diluted by acetonitrile, respectively. The stock solution and intermediate solution were kept at −80 °C. The series of gradient mixture standard working solutions (0.1, 0.5, 1, 5, 10, 25, 100, 500 μg L^-1^) and the quality control (5, 25, 100 μg L^-1^) samples were further prepared using Ringer’s solution (147 mM Na^+^; 2.2 mM Ca^2+^; 4 mM K^+^; pH 7.0, Shanghai yuanye Bio-Technology Co., Ltd.) and stored at −20°C.

### 2.2 Animal and treatment

SPF-grade male Sprague Dawley (SD) rats (220 ± 20) g were purchased from Henan Huaxing Experimental Animal Center (License number SCXY(Yu) 20190002, Zhengzhou, China). All rats were housed under a stable condition with *ad libitum* access to food and water under a 12 h light-dark cycle. Animal studies and experimental procedures were all strictly conducted under the condition of permission and supervision.

To inhibit the main Nic metabolic enzyme CYP2B in the brain, rats received intraperitoneal injections of proadifen (50 mg kg^−1^·day^−1^, 4 d) (Song et al., 2012). Control animals were pretreated with saline using the same protocol. All the rats were sacrificed by cardiac perfusion after microdialysis sampling. The brain tissues were separated and divided into four parts (cerebrum, cerebellum, diencephalon, and brainstem). The CYP2B activity in different rat brain regions was determined following the instructions of the GENMED CYP2B1 activity fluorescence quantitative detection kit (GENMED SCIENTIFICS INC. U.S.A, GMS18019.1).

### 2.3 Microdialysis sampling

Anesthesia was induced with 3% isoflurane in an induction chamber before surgical procedures and was placed in a stereotaxic frame equipped with a rat ear and bite bar. Rat was maintained under anesthesia with 1%–2% isoflurane during operation procedures. And the cranium above the ventral striatum (coordinates relative to bregma: anterior/posterior (*AP*), +1.2 mm; medial/lateral (*ML*), −2.4 mm; dorsal/ventral (*DV*), −6.0 mm) or NAc (coordinates relative to bregma: anterior/posterior (*AP*), +1.7 mm; medial/lateral (*ML*), −1.5 mm; dorsal/ventral (*DV*), −6.5 mm) was exposed to implant the probe guide-cannula (CMA/12), two screws were implanted into the skull to serve as an anchor and dental cement was used as an adhesive to fixed the probe guide-cannula. To avoid the interference of neurotransmitter changes due to physical stimulation of pain receptors during intraperitoneal injection of Nic, Nic was injected with the aid of an administration catheter (Instech, BTPU-040, 6.35 × 10.16 mm, America), which was implanted in the abdomen, the other end of the catheter was passed under the skin and sent to the back and sealed with a plug (Instech, PNP3M-F22R, America). The rats were housed individually and allowed to recover from surgery for at least 24 h.

For *in vivo* microdialysis sampling, the probe (CMA/12 Elite, membrane parameters: polyarylethersulfone material; 2 mm length; φ 0.5 mm; 20 kDa cut-off) was inserted into the brain through the cannula. After implantation, the probe was perfused with Ringer’s solution by the syringe pump (CMA/402, piston propulsion speed: 2.4 μm·min^-1^–1.2 mm min^-1^; velocity range: 0.1 μL·min^-1^–20 μL min^-1^; precision: −1.5%-1.5%) at fixed flow rates (1.5 μL min^-1^), and the dialysates were analyzed under the optimized UHPLC-HRMS/MS condition directly. Intraperitoneal administration of 2 mg kg^-1^ Nic was performed until stable levels of neurotransmitters were obtained, which was defined as the mean value of neurotransmitter concentrations in six consecutive samples varied by no more than 10%.

### 2.4 Chromatographic conditions and mass spectrometry settings

UHPLC-HRMS/MS system was performed on Dionex Ultimate 3000 UHPLC equipped with a Q-Exactive mass spectrometer and electrospray ionization (ESI) interface (Thermo Fisher Scientific, Germany). Chromatographic separation was achieved by the pentafluorophenyl phase column (Discovery^®^ HS F5-3 column, 2.1 × 150 mm, 3 μm) with linear binary gradient elution, at a flow rate of 0.5 mL min^-1^. Eluent A was 10 mM ammonium acetate with FA (0.1%, *v/v*) dissolved in water, and eluent B was acetonitrile with 0.1% FA (*v/v*). The gradient elution was started at 10% B and kept for 1 min, then linearly increased to 45% B over 2 min. From 3 to 6 min, a linear gradient was applied from 45% to 75% B and then return linearly to the initial condition at 9 min and the column was allowed to equilibrate for 1 min.

The mass spectrometer was operated in positive ionization mode. The optimized mass spectrometer parameters were as follows: a resolution of 35000; automatic gain control (AGC) target was 2.0 e^5^; maximum injection time (IT) was 100 m; 3.5 kV for spray voltage; ion transfer tube temperature, 350°C; S-Lens RF level, 60%. The isolation window of the precursor ions was done using a quadrupole with an isolation window of m/z 1. The normalized collision energy was set at 10%. Analytes were determined by a parallel reaction monitoring (PRM) method by acquiring two transitions for each compound. The most specific transition was selected for the quantitative purpose. All data were gathered and analyzed using Xcalibur 2.2 software (Thermo Fisher Scientific, Germany).

## 3 Results

### 3.1 Establishing the method for *in vivo* sampling and simultaneous online detection

**FIGURE 1:**
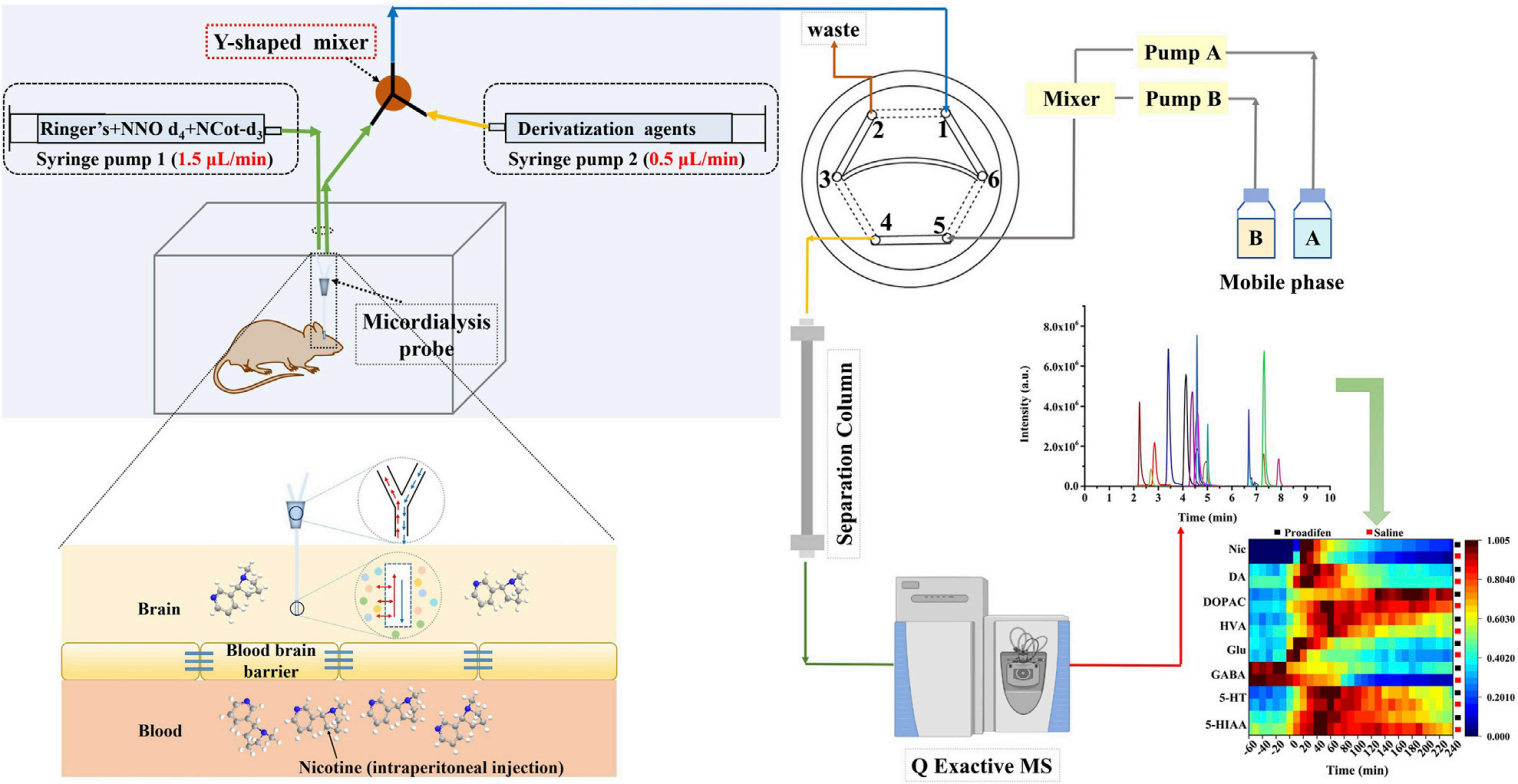
Schematic diagram of the precolumn derivatization online microdialysis UHPLC-HRMS/MS system.

In order to improve the response of several monoamine derivatives and reduce the differences in properties between Nic metabolites and certain neurotransmitters, we modified the conventional microdialysis system by adding a Y-shaped in-house binary static mixer at the outlet of the microdialysis probe to combine the derivatization reagent with dialysate online. The multi-step derivatization reaction condition reported in the literature (Wong et al., 2016) was modified to complete the reaction in one step, which was necessary to realize the *in-situ* derivatization system. The derivatization reagent was composed of 100 mM sodium carbonate and benzoyl chloride (10% (*v/v*) in acetonitrile) at 3:1 (*v/v*), and the pH was adjusted to 9.2 with sodium hydroxide. The system consisted of two syringe pumps for supplying Ringer’s solution at a flow rate of 1.5 μL min^-1^ and the derivatization reagent at a flow rate of 0.5 μL min^-1^, respectively. The derivatized extracellular fluid from microdialysis was introduced into the UHPLC-HRMS/MS system through an online injector (six-port injection valve) to realize the direct analysis, which minimized the possible contamination and degradation during sample storage and transfer. Specifically, port 1 was used to sample the derivatized dialysate, and port 3 and port 6 were connected through a 20 μL quantitative loop for loading the sample **(** Figure 1 **)**. The mobile phase was pumped into port 5, and port 4 was connected to the chromatographic separation column. The injection time difference between run 1 and run 2 was the separation time of run 1; that is, the solid line in the diagram represents the loading state lasting 10 min, and the dashed lines indicate the injection state for 0.2 min, so that as soon as run 1 ended, run 2 began immediately, and this cycle was repeated to obtain uninterrupted *in vivo* data.

**FIGURE 2:**
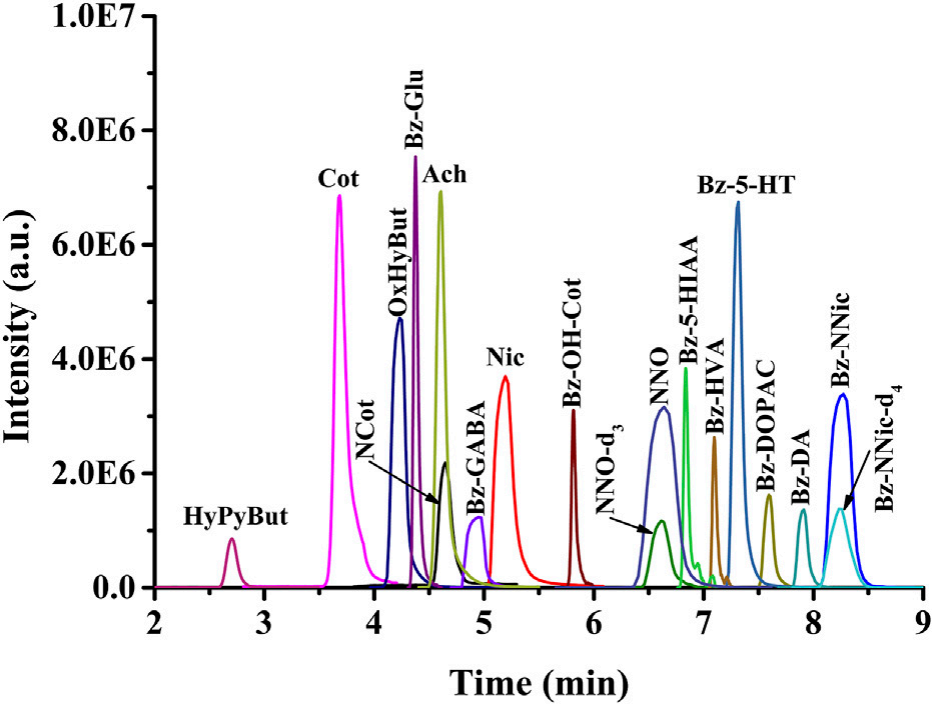
Representative PRM chromatograms of target analytes.

Despite the high specificity of the HRMS/MS detector, the electrospray ionization technique is susceptible to interference by co-eluting compounds; therefore, suitable chromatographic separation and an appropriate retention time were essential to improve the quantitative accuracy. To achieve better separation and sharper peaks of analytes in a short time, different chromatographic columns, mobile phases (acetonitrile-H_2_O, methanol-H_2_O), and salt concentrations were investigated. The final test results indicated that the pentafluorophenyl phase column was the best choice; that 0.1% formic acid should be added to both phases to provide an acidic environment; and that 10 mM ammonium formate was helpful for improving the peak shape. The gradient elution program was adjusted so that the target compounds were well separated from 2 min to 9 min **(Figure 2** **)**, and the chromatographic column can completely return to the initial pressure at 10 min for the next online injection. The suitable separation conditions and the matched temporal resolution of microdialysis sampling provided an advantage for correlating the relationship between Nic metabolism and its neurochemical effects.

Among the selected compounds, NNic, NCot, OH-Cot, and several neurotransmitters were benzoylated. For the best ion response, MS parameters, including the spray voltage, capillary temperature, vaporizer temperature, sheath gas, auxiliary gas, and S-lens RF level were manually tuned. PRM mode was used to provide the highest specificity and sensitivity. The mass accuracy between theoretical and experimental masses of all analytes was less than 2 ppm (Table 1). Based on the good separation ability of the UHPLC column and high accuracy of the Q-Orbitrap HRMS, the high selectivity of the method was guaranteed.

**TABLE 1:** Accurate mass and PRM parameters of the analytes.

Analyte	Protonated ion mass		Mass error (ppm)	nCE (%)	Main daughter ion (m/z)^a^	MS species
	Theoretical	Experimental				
Nic	163.1230	163.1231	0.6	55	**130.0652**/132.0808	M + H^+^
Cot	177.1022	177.1021	−0.5	70	**80.0501**/98.0604	M + H^+^
mono-Bz-NNic	253.1335	253.1331	−1.5	55	**147.0916**/235.1232	M + H^+^
mono-Bz-NNic-*d*_4_	257.1587	257.1584	−1.1	30	**105.0338**	M + H^+^
NCot	163.0866	163.0865	−0.6	70	**80.0500**	M + H^+^
NNO	179.1179	179.1176	−1.6	55	**132.0807**/130.0650	M + H^+^
NNO-*d*_3_	182.1367	182.1368	0.5	55	**132.0807/**130.0650	M + H^+^
CNO	193.0972	193.0971	−0.5	65	**96.0449**/98.0605	M + H^+^
mono-Bz-OH-Cot	297.1234	297.1235	0.3	25	**175.0864**/279.1121	M + H^+^
OxPyBut	180.0655	180.0657	1.1	50	**134.0603**	M + H^+^
HyPyBut	182.0812	182.0811	−0.5	50	**164.0705**/109.0525	M + H^+^
tri-Bz-DA	466.1649	466.1647	−0.4	15	241.0859/**105.0338**	M + H^+^
bi-Bz-DOPAC	394.1285	394.1283	−0.5	25	**105.0338**	M + NH_4_^+^
mono-Bz-HVA	304.1179	304.1182	0.9	10	137.0599/**105.0338**	M + NH_4_^+^
bi-Bz-5-HT	385.1547	385.1545	−0.5	25	**264.1018**/105.0338	M + H^+^
mono-Glu	252.0867	252.0863	−1.6	10	**105.0338**	M + H^+^
mono-GABA	208.0968	208.0969	0.4	10	**105.0338**	M + H^+^
mono-Bz-5-HIAA	296.0917	296.0912	−1.6	15	146.0599/**105.0338**	M + H^+^
Ach	146.1176	146.1175	−0.6	35	**87.0446**	M + H^+^

^a^ Quantification ion transitions are in bold.

### 3.2 Method validation

NNic-*d*_*4*_ and NNO-*d*_*3*_ were added to the perfusate and used as internal standards after retrodialysis for derivatized and non-derivatized products, respectively, which eliminated the minor errors caused by instrument analysis and corrected the small deviation of the dialysis rate between samples during the long sampling process of microdialysis. The instrument analytical performance was investigated in terms of linearity, precision and accuracy, limit of detection (LOD), and limit of quantification (LOQ) under the optimized conditions. The developed method had a wide range for the Nic metabolites and neurotransmitters with the coefficient of determination (*R* ^2^) higher than 0.9935 for all analytes. The LOD and LOQ were from 0.003 to 1.0 μg L^-1^ (*S/N* = 3) and from 0.01 to 2.5 μg L^-1^ (*S/N* = 10), respectively. The intra-day precision and accuracy ranged from 1.7% to 8.4% and −2.6% to 6.5%, respectively (Table 2). All these results were within the acceptable criteria, indicating that the established method was reliable and suitable for the simultaneous analysis of Nic metabolites and neurotransmitters in microdialysis samples.

**TABLE 2:** Linearity, accuracy, precision, limits of detection (LODs) and microdialysis recoveries for target analytes.

Analyte	LOD (μg·L^-1^)	Linear range (μg·L^-1^)	*R* ^2^	Accuracy (RE, %) (n = 5, μg·L^-1^)			Precision (RSD, %) (n = 5, μg·L^-1^)			*R*_dial_ of probe
				5	25	100	5	25	100	
Nic	0.3	1.0–250	0.996	2.5	1.3	3.5	5.3	4.1	3.2	30.0 ± 3.6
Cot	0.003	0.01–250	0.998	1.5	3.2	2.7	2.2	3.0	4.1	21.3 ± 4.7
Bz-NNic	0.03	0.1–250	0.992	2.2	0.5	1.9	3.2	5.7	1.7	18.8 ± 2.5
NCot	0.003	0.01–250	0.998	1.5	1.3	3.0	2.4	2.5	3.7	18.1 ± 3.1
NNO	0.3	1.0–250	0.997	2.1	0.9	1.9	3.7	7.1	4.4	22.8 ± 2.0
CNO	0.015	0.05–250	0.995	2.1	−1.8	0.9	3.6	7.5	5.5	25.8 ± 3.0
Bz-OH-Cot	0.15	0.5–250	0.995	1.2	0.6	1.9	4.2	2.2	3.6	21.3 ± 2.6
OxPyBut	0.03	0.1–250	0.999	2.5	0.8	1.5	2.9	2.7	3.1	23.3 ± 1.6
HyPyBut	0.03	0.1–250	0.999	2.8	3.8	2.1	3.8	4.1	4.2	22.3 ± 2.9
Bz-DA	1.0	2.5–500	0.994	6.5	3.3	2.9	8.4	6.8	7.4	20.7 ± 4.2
Bz-DOPAC	0.3	1.0–500	0.996	4.5	5.8	5.3	5.3	5.3	7.9	31.8 ± 2.5
Bz-HVA	0.3	1.0–500	0.996	6.4	4.0	4.7	7.1	6.2	7.5	18.1 ± 1.1
Bz-5-HT	1.0	2.5–500	0.999	6.1	4.0	3.5	7.0	5.3	6.4	28.0 ± 1.0
Bz-5-HIAA	0.03	0.1–500	0.996	1.4	2.4	3.6	1.9	3.9	4.6	25.3 ± 1.8
Bz-GABA	0.30	1.0–500	0.996	3.7	2.9	1.0	7.0	7.2	2.8	20.4 ± 2.1
Bz-Glu	0.3	1.0–500	0.997	4.3	−2.6	0.8	4.8	3.8	2.7	19.1 ± 3.0
Ach	0.3	1.0–500	0.995	2.6	2.4	0.7	5.7	2.7	4.2	22.1 ± 1.9

### 3.3 Determination of Nic metabolites and neurochemicals in rat brain

To demonstrate the utility of the method in monitoring the low concentration analytes in the dialysates, rats were exposed to Nic by single intraperitoneal injection (2 mg kg^-1^, i. p.), and the brain samples from the ventral striatum were analyzed as extracted ion chromatograms. All the expected Nic metabolites in the dialysate were detected, and NCot was only detected in a few periods. The extracted ion chromatograms of Nic metabolites and classical neurotransmitters are shown in Supplementary Figure S1. In addition, another six amino acids (aspartic acid, glycine, glutamine, histidine, taurine, and serine) were incidentally extracted, which were confirmed by the precise molecular weight of the derivatized product (238.0710, 180.0655, 251.1026, 260.1029, 230.0482, and 314.1023, respectively) and benzoyl fragment (m/z 105.0338) (Supplementary Figure S2). These results suggested that the developed precolumn derivatization-based online microdialysis coupled with UHPLC-HRMS/MS was not just a proof-of-principle study. It not only fulfilled the need to investigate the release of neurotransmitters during Nic metabolism, but simultaneously also monitored the changes of other neurochemicals, which may shed more light on the neurobiological mechanisms underlying behavioral changes induced by Nic exposure.

We further investigated the time course curves of Nic metabolites and monoamine neurotransmitters in rat striatum after administration of Nic (2 mg kg^-1^, i. p.) (Supplementary Figure S3). The results showed that the most abundant Nic metabolites in rat striatum were Cot, NNO, and NNic, and the brain metabolic process of Nic was similar to that in the peripheral system. The rapid distribution of Nic in the brain induced a high intensity release of monoamine neurotransmitters. After 3 h of Nic exposure, the average levels of DA and its metabolites DOPAC and HVA, and 5-HT and its metabolite 5-HIAA were 25.1, 99.2, 169.8, 45.9, and 234.4 μg L^-1^, respectively. It is worth noting that the levels of DA and 5-HT were lower than their metabolites, which showed that neurotransmitters were rapidly metabolized or absorbed after being released into the synaptic space.

### 3.4 Effect of proadifen pretreatment on the metabolism of Nic in rat brain

**FIGURE 3:**
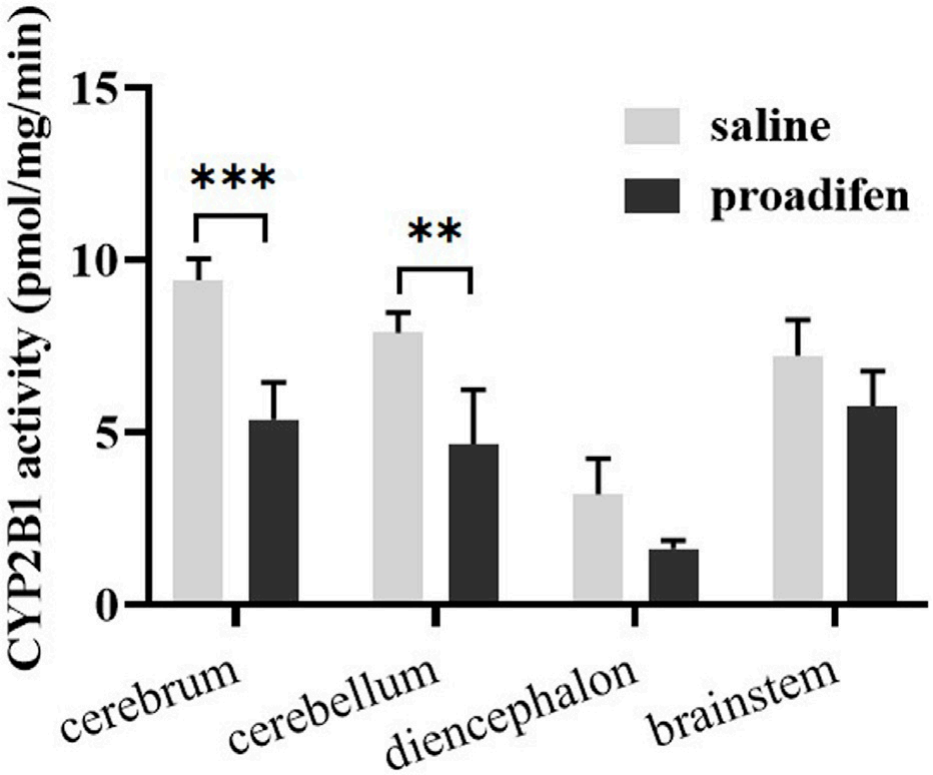
The activities of CYP2B1 in different parts of brain tissue (data are expressed as mean ± SD from three rats). Significant differences were determined by two-way ANOVA and Tukey’s post-hoc test. ***p < 0.001, **p < 0.01.

**FIGURE 4:**
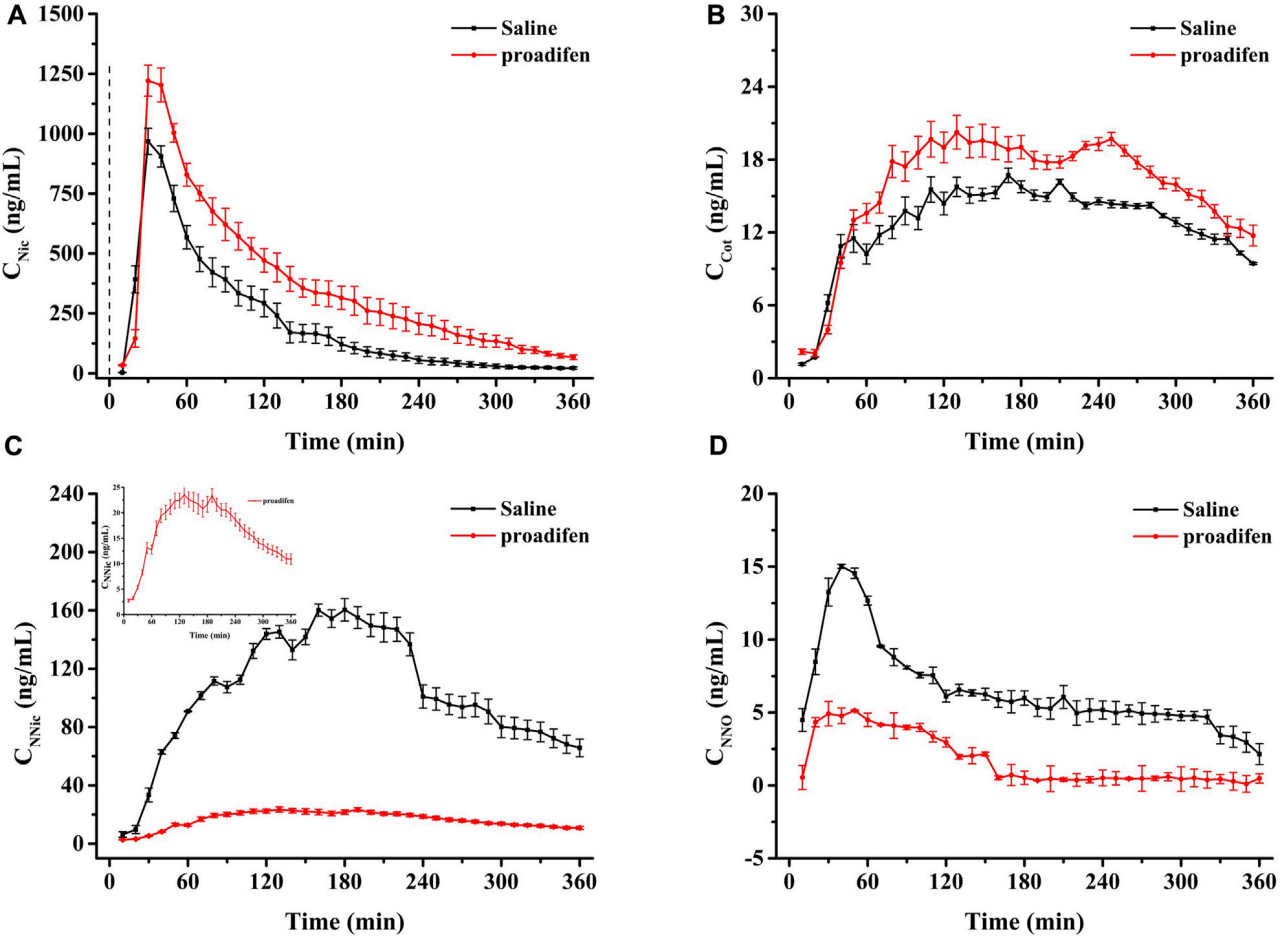
Time-course curves of Nic **(A)**, Cot **(B)**, NNic **(C)**, and NNO **(D)** in rat brain after administration of Nic (2 mg kg^-1^, i. p.) (Each point was expressed as mean ± SD from three rats).

CYP2B1, which corresponds to human CYP2B6, plays a major role in the metabolism of Nic in rat (Hammond et al., 1991; Al Koudsi and Tyndale, 2010). Previous studies using rats confirmed that alternating brain CYP2B activity influenced brain Nic levels and Nic-mediated behaviors (Garcia et al., 2015; Garcia et al., 2017). To investigate the effect of CYP2B inhibition on Nic biotransformation and neurotransmitter release in rat brains, another group of rats was pretreated with proadifen (50 mg kg^-1^·d^-1^, i. p., 4 days) in our research. Compared with the saline-pretreated control group, the activities of CYP2B1 were inhibited in different brain tissues, including the cerebrum, cerebellum, diencephalon, and brainstem (Figure 3). The whole brain CYP2B1 activity decreased by 36.9% on average after proadifen treatment. The brain concentration–time profiles of Nic and its main metabolites Cot, NNO, and NNic were generated using the mean concentration levels of each time bin (Figure 4), and the pharmacokinetic parameters of each analyte estimated by the two-compartmental model are listed in Table 3. The concentration of Nic increased rapidly after intraperitoneal injection and then gradually declined. By 360 min, Nic was almost completely cleared from the rat brain. There was no difference in the *t*_max_ of Nic between the proadifen-pretreated rats and the saline-pretreated group. However, the levels of Nic in the proadifen-pretreated rats were consistently higher than those of the saline group between *t*_max_ to 360 min. The *C*_max_ of Nic in the proadifen group was as high as 1221 μg L^-1^, which was 1.26 times higher than that in the saline group. The greater *t*_1/2_ and mean residence time (MRT) values indicated a slower elimination, which could also be explained by the increase of the area under the curve (AUC) and was consistent with Tyndale’s study. Compared with the control rats exposed to Nic, the *C*_max_ of NNic and NNO decreased by 85.3% and 65.6% after CYP2B1 inhibition, respectively.

**TABLE 3:** Estimated pharmacokinetic parameters for Nic and its main metabolites from rat NAc treated with single injection of Nic (2 mg kg^-1^, ip).

	AUC_0–360 min_ (mg·min^-1^·mL^-1^)		CL/F (mL min^-1^)		*t*_1/2z_ (min)		MRT (min)		*t*_max_ (min)		*C*_max_ (μg L^-1^)	
	Saline	Prodifine	Saline	Prodifine	Saline	Prodifine	Saline	Prodifine	Saline	Prodifine	Saline	Prodifine
Nic	75.1	131.5	25.0	14.0	128.8	76.7	85.9	113.5	30	30	968.3	1221.3
Cot	4.6	5.6	9.0	7.0	104.3	131.1	186.7	187.1	160	120	16.7	20.3
NNic	37.4	5.9	2.0	7.0	115.9	166.9	179.9	179.4	170	120	160.4	23.5
NNO	2.3	0.6	5.0	3.2	48.1	53.7	143.3	94.5	30	40	15.1	5.2

### 3.5 The release of neurotransmitters in the NAc under the regulation of Nic metabolism

**FIGURE 5:**
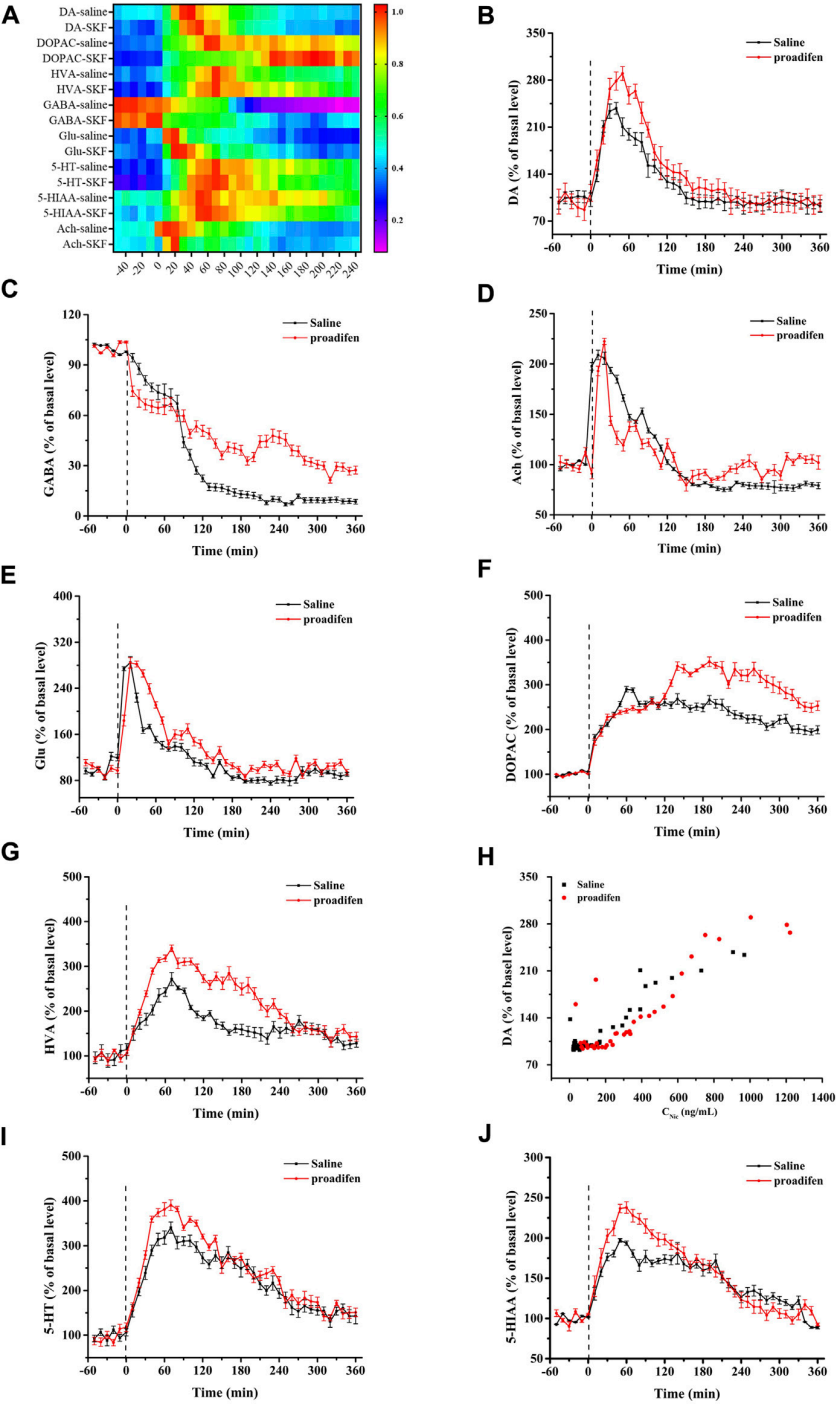
**(A)** The heat map shows all changes of neurotransmitters, where the colors correlated with the changes expressed as percentage of baseline. Time course of Nic exposure evoked DA **(B)**, Glu **(C)**, Ach **(D)**, GABA **(E)**, DOPAC **(F)**, HVA **(H)**, 5-HT **(I)**, and 5-HIAA **(J)** release in the NAc, data were expressed as % of basal release. (Each point was expressed as mean ± SD from three rats) **(G)** Scatter plot of Nic concentration and DA release level.

To eliminate the differences in neurotransmitter levels between each rat, the changes of all neurotransmitter levels were expressed as the relative values of neurotransmitter concentrations for each time bin to baseline values before Nic exposure. Nic yielded a series of complex changes in the classical neurochemicals in the NAc (Figure 5A). It induced an increase in baseline levels of DA, Glu and Ach (Figures 5B–D), but caused almost the opposite change in the inhibitory neurotransmitter GABA (Figure 5E). The peak concentration of the DA metabolites DOPAC and HVA reached nearly 290% and 270% in the saline group, and 340% and 330% of the baseline in the proadifen pretreated group after Nic stimulation (Figures 5F, G), respectively. The peak level of Ach after Nic injection was almost doubled of its baseline levels, but proadifen inhibition did not further increased it. The level of GABA in the proadifen group was 2.7 times higher than that in the saline group on average. The trend of the DA curve appeared to mirror the concentration-time curve of Nic, and the concentration of Nic and DA release was positively correlated (Figure 5H). The levels of 5-HT and its metabolite 5-HIAA in the rat NAc first increased and then decreased slowly after Nic exposure. The *C*_max_ of 5-HT and 5-HIAA were almost 390% and 240% of baseline in the proadifen pretreatment group, respectively (Figures 5I, J).

## 4 Discussion

As a major reinforcing ingredient of tobacco products that is responsible for addiction in smokers, Nic has multiple and complex effects, such as reward, analgesia, and an improvement in mood or cognitive function. All these effects stem from the presence of Nic in the brain, which triggers a cascade of downstream signaling events by binding to nAChRs, and mainly modulates multiple neurotransmitter systems. The continual neuropharmacological effects of Nic are closely related to the brain tissue distribution and the body’s metabolic ability. However, information on the detailed neurotransmitter release induced by Nic during its metabolic process has rarely been reported, which is an obstacle to comprehensively elucidating its complex physiological effects. Even though the peripheral pharmacokinetics of Nic have been extensively studied, these studies cannot truly reflect the local biotransformation of Nic in the brain. Therefore, it is necessary to synchronously investigate the brain disposition, metabolic characteristics, and the related neurotransmitter effects resulting from Nic exposure in the CNS.

Synchronous investigation of brain Nic pharmacokinetics and the changes of various neurotransmitters has been given little attention because of the difficulty of continuously obtaining the brain samples *in vivo* and the lack of an effective detection method. The local biotransformation of Nic in the brain has been studied in our recent work, and approximately 10 Nic metabolites have been determined by brain microdialysis coupled with UHPLC-HRMS/MS (Xu et al., 2019). Many assays for the detection of classic neurotransmitters from brain samples have been developed, such as electrochemical detection, HPLC-fluorescence detection, and HPLC-MS. Although continuously obtaining the animal brain samples *in vivo* could be solved by microdialysis techniques, simultaneously determining Nic metabolites and various neurotransmitters using HPLC-MS/MS is challenged by the disparate polarity of the molecules, trace levels in the brain, and the extremely unstable chemical properties of some neurotransmitters (Nandi and Lunte, 2009; Jha et al., 2018; Olesti et al., 2019; Zhou et al., 2020). In this work, precolumn derivatization-based online microdialysis coupled with UHPLC-HRMS/MS was successfully developed to monitor the changes of Nic metabolites and several neurochemicals. This achieved minimally invasive continuous sampling of awake animals and avoided degradation and contamination caused by sample preservation and transfer. Using this approach, Nic and its metabolites, five monoamine neurotransmitters, and eight amino acids were simultaneously obtained in rat striatum dialysate following Nic peripheral exposure (2 mg kg^-1^, i. p.). Compared with the G-protein coupled receptor-based sensors for imaging neurochemicals developed by Li et al. and *in vivo* enzymatic electrochemical biosensors for *in situ* neurochemical measurements in recent years (Liu et al., 2017; Jing et al., 2019; Pan et al., 2020; Sun et al., 2020), although the temporal resolution based on our analysis was poor, it allowed us to achieve long-term and simultaneous monitoring of several compounds, including neurotransmitters. It is not possible with these other novel technologies to investigate the metabolic effects of Nic on multiple neurotransmitter systems.

Variations in both the levels of Nic in the brain (via Nic metabolizing enzymes) and the brain response to Nic (via nAChRs binding) may influence the multiple pharmacological properties of Nic. To investigate the effect of the Nic metabolic rate on neurotransmitter release in the NAc, which is innervated by DAergic neurons of the mesolimbic reward system, rats were pretreated with proadifen, and the CYP2B activity in the brain decreased by 36.9% compared with the saline group. The *N*-demethylation and *N*-oxidation metabolic pathways of Nic were measurably downregulated in the brain compared to the saline-treated control, and the *C*_max_ and AUC of the *C*-oxidation product Cot were lower in the saline and proadifen group, indicating that CYP2B plays a key role in the *N*-demethylated and *N*-oxidated biotransformation of Nic in the rat NAc. In addition, proadifen pretreatment showed no effect on the *t*_max_ of Nic, which indicated that inhibition of CYP2B did not affect the rate of Nic entry into the brain, as studies have shown that the absorption rate of Nic largely depends on the molecular forms of Nic (Hukkanen et al., 2005).

The release and regulation of neurotransmitters between hundreds of millions of neurons play a vital role in maintaining normal physiological functions of the body. Much evidence has indicated that Nic binds to α4β2 and α7 nAChRs located on DAergic, glutamatergic, and GABAergic neurons in the mesolimbic DA system, which is responsible for the increase in extracellular DA in the NAc (Xi et al., 2009), but as a strong agonist of nAChRs, it is little known about the effects on extracellular Ach release in the NAc. We found that acute Nic treatment affects the release and metabolism of DA in rat NAc. The levels of DA and its metabolites, such as DOPAC and HVA, were elevated, which was consistent with Pietilä’s results that DOPAC and HVA were significantly increased and 3-MT was decreased after acute Nic administration in striatum (Pietilä et al., 1996). The level of DA in the rat NAc increased after intraperitoneal injection of Nic, and the level of DA metabolites, DOPAC and HVA, were higher than DA, which confirmed that DA was rapidly metabolized after release into the synaptic cleft. The higher concentration of Nic after proadifen inhibition enhanced the spike of DAergic neurons in the VTA, which thereby increased the release of excitatory neurotransmitters in the NAc. Like Nic, Ach is also involved and binds with nAChRs. However, little knowledge about the effects of Nic on extracellular Ach release in the NAc. The basal forebrain contains large number of acetylcholinergic neurons, and the NAc is mainly composed of GABAergic neurons. However, we found that after Nic stimulation, Ach in the NAc also increased sharply, this may be due to the regulation of NAc by cholinergic neurons in the prefrontal cortex (Cooper and Henderson, 2020), and some research has reported that Ach in the NAc is associated with aversive states and satiation (Rada et al., 2001; Hoebel et al., 2007). In addition, Nic acted on the nAChRs on GABAergic neurons in NAc, and nAChRs were desensitized by the high Nic concentration, which decreased the GABAergic effects on DAergic neurons, resulting in the disinhibition of DA neurons (Grieder et al., 2019; Cooper., et al., 2020). These results also speak to the previous report that DAergic neurons in the VTA project to GABAergic and cholinergic interneurons in the NAc, which together with glutamatergic projections from the prefrontal cortex provide a complex regulatory network with multiple effects of Nic (Morozova et al., 2020). Studies have shown that compared with smokers with a normal Nic metabolism ability, individuals lacking full functional CYP2A6 were perceptibly protected against becoming tobacco-dependent smokers (Pianezza et al., 1998). Nic plus methoxsalen given orally inhibits the first-pass metabolism of Nic, and the combination directly reduces the desire to smoke (Sellers et al., 2000). These results indicated that the decrease of Nic metabolism caused by genetic or drug factors can alleviate dependence-related behaviors. Our present results extend these findings, demonstrating that the influence of inadequate Nic metabolism on related behaviors is due to the relatively high level and long residence of Nic in the brain that can trigger a more lasting release of neurotransmitters.

After inhibiting the metabolism of Nic, higher concentrations of Nic in the NAc continued to act on 5-HTergic neurons to exert an analgesic effect. This may be related to the anti-nociception properties of Nic confirmed by extensive and compelling studies that found that peripheral administration of methoxsalen for inhibiting mouse Nic metabolism dramatically prolonged the Nic-induced analgesic effect, which was confirmed by tail-flick and hot-plat tests. In addition, Shen et al. established a rat Nic withdrawal model and founded that after Nic discontinuation, the mechanical withdrawal threshold and thermal withdrawal latency of rats were notably reduced with the decreased level of 5-HT; however, intrathecal injection of 5-HT abolished these differences between the control and Nic withdrawal group (Shen et al., 2021). Based on these related behavioral phenomena reported previously and the results of our analysis, it was further indicated that 5-HTergic neurons may play a key role in the analgesic effect of Nic.

In conclusion, correlating the changes of neurotransmitters with brain Nic levels during its metabolic process in the body can provide more definitive evidence for comprehensively understanding the influence of Nic clearance on its neuropharmacological actions. Our results indicated that the precolumn derivatization-based online microdialysis coupled with a UHPLC-HRMS/MS system was an efficient and novel technique to synchronously monitor the brain Nic metabolism and neurochemical changes in freely-moving animals. After inhibition of the rat CYP2B activity, the brain Nic pharmacokinetics changed, and the higher concentration of Nic in the brain led to a further release of the excitatory neurotransmitter DA, as well as the release of the inhibitory neurotransmitter GABA, which may be one of the reasons why the high concentration of Nic would produce adverse effects. The increased 5-HT release further confirmed the previously reported phenomenon that the analgesic effect of Nic was prolonged after inhibiting Nic metabolism.

## 5 Conclusion

We developed a precolumn derivatization-based online microdialysis coupled with UHPLC-HRMS/MS method to achieve continuous sampling and simultaneous investigation of Nic metabolites and several neurochemicals in the brain. The usefulness of this platform was confirmed by monitoring the brain pharmacokinetic profiles of Nic and the associated time-dependent neurotransmitter release following peripheral Nic exposure. The *N*-demethylation and *N*-oxidation metabolic pathways of Nic were significantly declined in the brain with the inhibition of rat CYP2B activity, inducing the higher level and longer residence of Nic and the enhanced release of neurotransmitters in the NAc. These results indicated that the disposition and metabolism of Nic was an important determinant of its neurochemical effects in the specific brain regions. To our knowledge, it was the first time to synchronously obtain the cerebral Nic pharmacokinetics and the related changes of neurotransmitters regulated by Nic metabolism. The present study provides a fundamental basis for monitoring the brain metabolic profiles and pharmacodynamics of Nic when developing the Nic delivery systems, Nic replacement therapy, or studying the potential role of Nic in improving depression and other neurological diseases.

## Data Availability

The datasets presented in this study can be found in online repositories. The names of the repository/repositories and accession number(s) can be found in the article/Supplementary Material.
