# Supplementary material for: Pharmacokinetic and pharmacodynamic studies of nicotine in rat brain: a simultaneous investigation of nicotine metabolites and the release of neurotransmitters in vivo
Source: Front Chem. 2023 Oct 23;11:1275478. doi: 10.3389/fchem.2023.1275478 (PMC10626537; doi:10.3389/fchem.2023.1275478)
Supplement: Supplementary file 1 [file DataSheet1.docx]

Supplementary Material

**Pharmacokinetic and pharmacodynamic studies of nicotine in rat brain: A simultaneous investigation of nicotine metabolites and the release of neurotransmitters *in vivo***

**Lulu Guo^1, 2, 3^†, Jian Mao^2, 3^†, Qidong Zhang^2^, Wu Fan^2^, Dingzhong Wang^2^, Zhonghao Li^2^, Jiaqiang Huang^1^, Jianping Xie^1,^ ^2, 3^***

^1^Beijing Advanced Innovation Center for Food Nutrition and Human Health, Department of Nutrition and Health, China Agricultural University, Beijing 100083, China

^2^Beijing Life Science Academy, Beijing 102209, China

^3^Food Laboratory of Zhongyuan, Zhengzhou University, Zhengzhou 450001, China

*** Correspondence:**
xiejian8065_cn@sina.com (Jianping Xie)

† These authors contributed equally to this work

# 1 Supplementary Figures


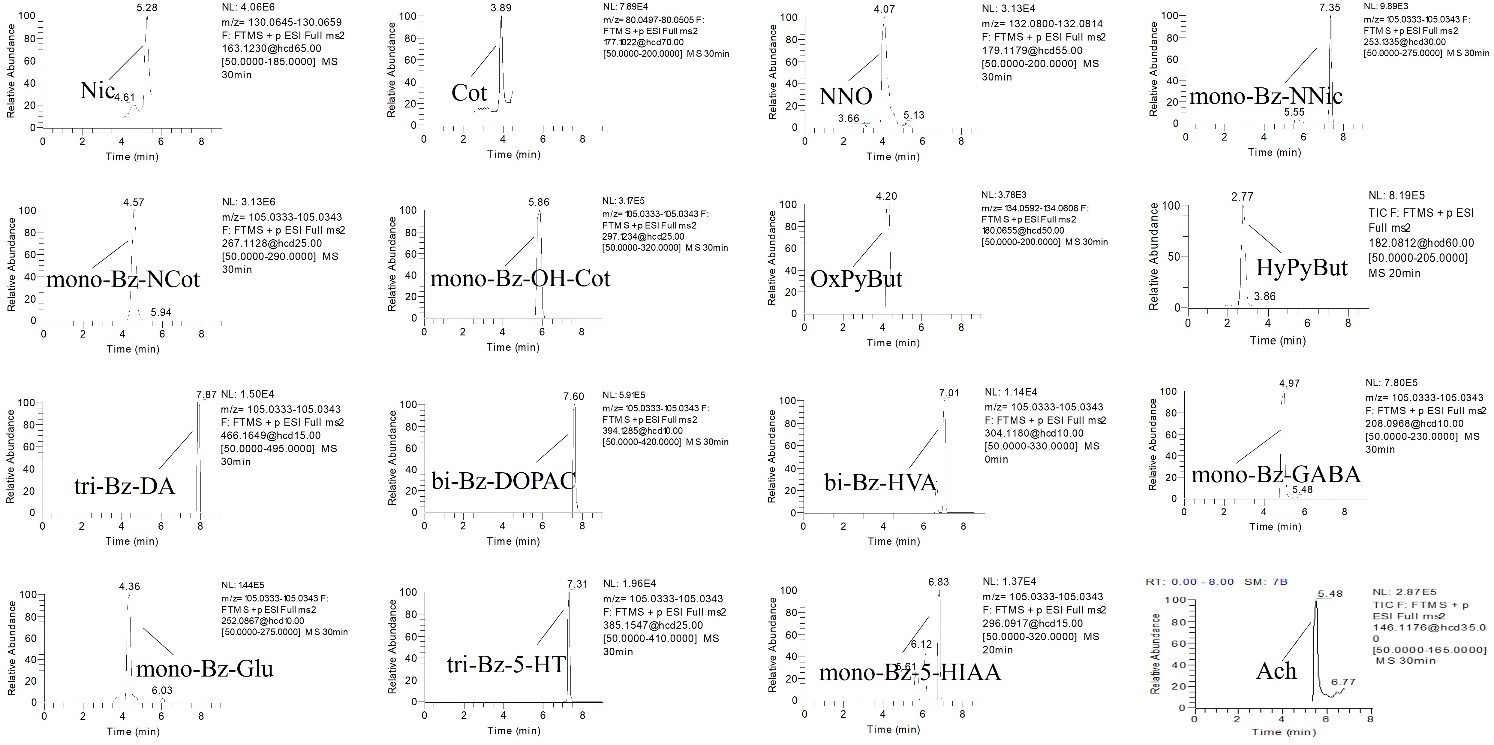


**Supplementary Figure 1.** Extracted ion chromatograms of Nic metabolites and monoamine neurotransmitters in rat striatum after administration of Nic (2 mg·kg^-1^, i.p.).


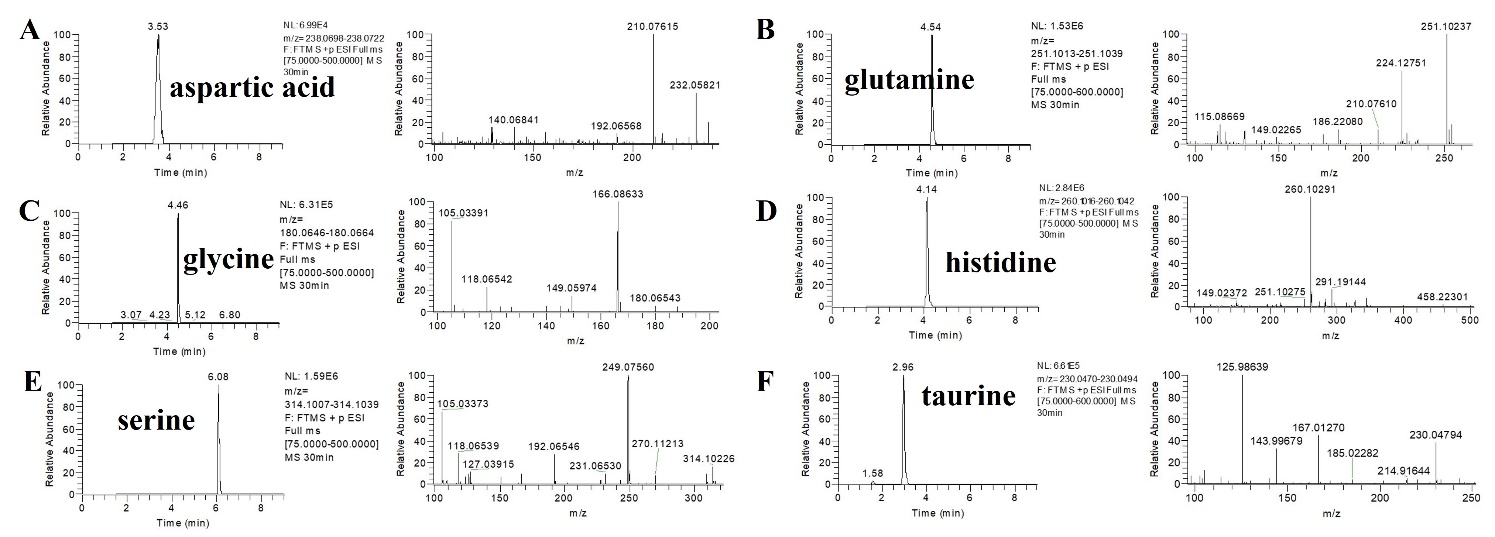


**Supplementary Figure 2.** Extracted ion chromatograms and mass spectra of amino acids in rat brain (glutamine: Gln, taurine: Tau, histidine: His, serine: Ser, aspartic acid: Asp, glycine: Gly).


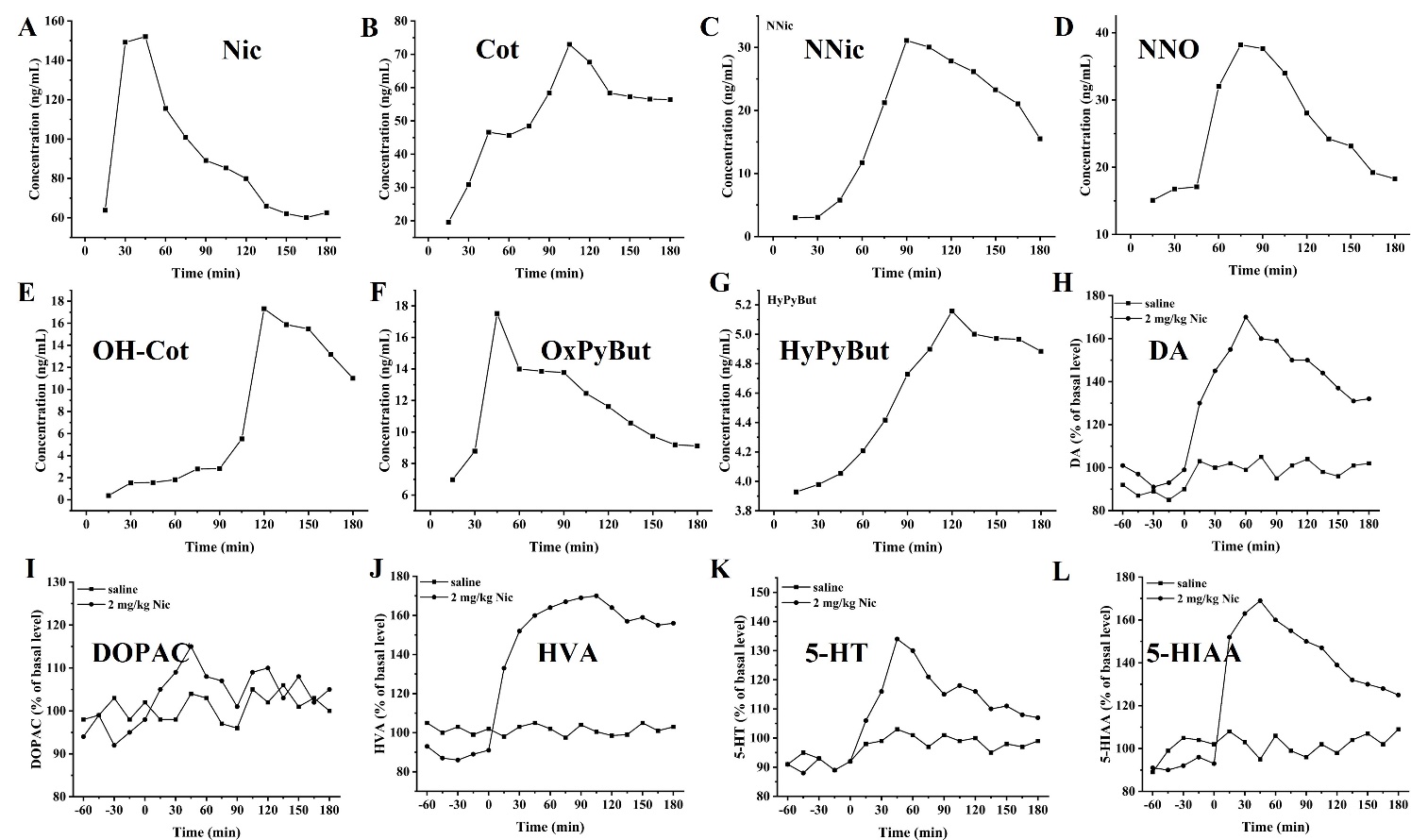


**Supplementary Figure 3.** The time-course curves of Nic metabolites and monoamine neurotransmitters in rat striatum after administration of Nic (2 mg·kg^-1^, i.p.).
